# Nearest Neighbour Node Deployment Algorithm for Mobile Sensor Networks †

**DOI:** 10.3390/s23187797

**Published:** 2023-09-11

**Authors:** Mahsa Sadeghi Ghahroudi, Alireza Shahrabi, Tuleen Boutaleb

**Affiliations:** School of Computing, Engineering and Built Environment, Glasgow Caledonian University, Glasgow G4 0BA, UK; mahsa.sadeghi@gcu.ac.uk (M.S.G.); t.boutaleb@gcu.ac.uk (T.B.)

**Keywords:** distributed mobile sensor network, node deployment algorithm, nearest neighbour, collective movement

## Abstract

Many animal aggregations display remarkable collective coordinated movements on a large scale, which emerge as a result of distributed local decision-making by individuals. The recent advances in modelling the collective motion of animals through the utilisation of Nearest Neighbour rules, without the need for centralised coordination, resulted in the development of self-deployment algorithms in Mobile Sensor Networks (MSNs) to achieve various types of coverage essential for different applications. However, the energy consumption associated with sensor movement to achieve the desired coverage remains a significant concern for the majority of algorithms reported in the literature. In this paper, the Nearest Neighbour Node Deployment (NNND) algorithm is proposed to efficiently provide blanket coverage across a given area while minimising energy consumption and enhancing fault tolerance. In contrast to other algorithms that sequentially move sensors, NNND leverages the power of parallelism by employing multiple streams of sensor motions, each directed towards a distinct section of the area. The cohesion of each stream is maintained by adaptively choosing a leader for each stream while collision avoidance is also ensured. These properties contribute to minimising the travel distance within each stream, resulting in decreased energy consumption. Additionally, the utilisation of multiple leaders in NNND eliminates the presence of a single point of failure, hence enhancing the fault tolerance of the area coverage. The results of our extensive simulation study demonstrate that NNND not only achieves lower energy consumption but also a higher percentage of k-coverage.

## 1. Introduction

In recent decades, there has been a significant interest in studying the spectacular phenomenon of animal collective movement. Such collective movement can be observed in various biological events, including the migration of bacteria [1,2,3], the flocking of fish and birds [4,5,6,7,8,9], and the coordinated motion of ants [10,11], whether in small or large groups. What makes these events truly fascinating is the distributed decision-making and leaderless movements of the individuals, resulting in coordinated behaviour. For example, the leaderless bird flock motion creates amazing patterns in the sky, which arise from the distributed decision-making processes of each bird. Understanding and analysing these collective movements can provide valuable insights for designing distributed decision-making systems. By developing models and algorithms inspired by the study of animal collective movements, it becomes possible to simplify the decentralized decision-making processes in complex systems, which can be used in various fields such as sensor networks, robotics, and more.

The collective movement of animals inspired many models [12,13,14,15,16]. One of the pioneering discrete models that exemplifies the collective behaviour of autonomous nodes is Vicsek’s algorithm [14]. This algorithm defines the motion of each self-propelled particle (SPP), such as a bird, by updating its location based on a local rule that takes into account the state of each particle and its neighbouring particles. This distributed decision-making process, known as the nearest neighbour rule, effectively emulates the collective movement observed in animal aggregations. Over the years, the nearest neighbour rule of Vicsek’s algorithm has been thoroughly studied and analysed by several researchers, providing valuable insights [15,17,18,19]. These studies have acted as a substantial source of inspiration for many innovative solutions and novel approaches in various research areas, including mobile sensor networks [19,20,21].

The analysis and modelling of collective movement in animal aggregations have had a profound impact on the development of decentralized control mechanisms for self-deploying mobile sensors [14]. In a network, mobile sensor nodes can emulate the individual particle behaviour observed in a collective movement to navigate within the network. A distinguishing feature of mobile nodes is their dynamic decoupling, meaning the movement of one individual node does not directly affect its neighbouring nodes [15,21,22,23]. This characteristic allows for the possibility of creating collective movement among mobile sensor nodes within a network, based on the nearest neighbour rule.

By employing the nearest neighbour rule, the collective movement within the network can converge all the mobile sensor nodes and guide them along the same trajectory based on their initial headings. The concept of convergence in mobile nodes over a period of time is stated in [22], while also considering limitations on the heading angles in some specific scenarios. Furthermore, the impact of introducing a static leader into a group of mobile sensor nodes and how the sensor nodes’ characteristics are aligned with the leader’s characteristics, are discussed in [17]. It was observed that including a static leader within a group of sensor nodes, operating under the nearest neighbour rule, leads to the convergence of their characteristics towards those of the leader. Consequently, by incorporating a static leader with adequate parameters, the sensor nodes in the network can be brought into alignment with those parameters, thus achieving convergence. Sensor nodes in a network can also be heterogeneous meaning leaders or any other sensor nodes in the network have different characteristics [24].

Building upon the findings presented in [15,20,21,23,25,26], a novel algorithm, which we refer to as Cheng algorithm, is presented in [22] to tackle the blanket coverage problem within mobile sensor networks. This algorithm utilises the nearest neighbour rule to guarantee the convergence of sensor nodes towards their respective assigned leaders within the group. This concept was initially explored in the context of barrier coverage with two leaders, aiming to synchronize the group of sensors between these leaders [26]. The exploration then extended to blanket coverage between two defined lines, where a single leader node is situated at the starting point of the left line to achieve complete coverage of the designated area. The proposed algorithm has been built from research on different types of coverage. Initially, a distributed self-deployment algorithm to cover a line is discussed [26]. Later, the coverage problem has been extended to barrier coverage and sweep coverage in corridor environment [20,27]. The decentralised control laws used in [20,27,28] are adopted to provide blanket coverage, full coverage of an area, in [21,23] and finally [22]. By employing the nearest neighbour rule, the sensor nodes converge towards the assigned static leader’s characteristics, while a control law restricts their movements within the defined boundaries. The resulting movement of the sensor nodes ensures that each sensor traverses the area in a sequential manner, facilitating full blanket coverage.

Although Cheng algorithm [21,22] provides full blanket coverage with no conflict between sensor nodes, it introduces a deterministic approach by establishing a singular stream of sensor motion to provide blanket coverage as the sensors are guided by one leader. It is important to note that this single-stream movement presents a potential single point of failure, as the entire system relies on the performance of a single leader. Additionally, the sequential movement of sensors throughout the entire area not only prolongs the deployment time but also significantly increases energy consumption. Finally, the Cheng algorithm may encounter issues with out-of-boundary movements, where the singular stream continues to operate beyond the predefined boundaries of the area, leading to increased energy consumption. Needless to say, this heightened energy consumption is of significant concern, especially for mobile sensors with inherently limited energy sources. Finding efficient strategies to reduce both the deployment time and energy consumption is therefore crucial in optimizing the performance and prolonging the operational lifespan of sensors.

In this paper, we propose a distributed node deployment algorithm, called Nearest Neighbour Node Deployment (NNND), to achieve blanket coverage in a mobile sensor network. Leveraging the advantages of parallelism, NNND facilitates the creation of multiple sensor motion streams, with each stream heading towards a different region of the designated area. To ensure cohesion within each stream and eliminate the risk of a single point of failure, a leader is adaptively elected for each stream. This utilisation of multiple leaders enhances fault tolerance and incorporates collision avoidance mechanisms. As a result, the algorithm effectively reduces the total distance travelled by sensors, thus minimizing energy consumption. It especially holds high importance as optimal coverage with a limited number of sensor nodes is a significant topic in industry and academia [29]. Furthermore, NNND incorporates control laws that prevent out-of-boundary movements. These control laws account for various features of the area, ensuring that sensor nodes remain within the predefined boundaries of the RoI.

The rest of the paper is organised as follows. Section 2 explains the preliminaries in two sub-sections, the area description in nearest neighbour algorithms and the assumptions of these algorithms. The NNND algorithm is explained in Section 3. The performance evaluation section to describe the simulation setup and results are under Section 4 and finally, the paper is concluded in Section 5.

## 2. Preliminaries

### 2.1. Area Description in Nearest Neighbour Algorithms

A Wireless Sensor Network (WSN) includes a set of sensor nodes $S=\{s_{1},s_{2},\cdots ,s_{n}|n\in N\}$, that provide the area coverage in a Region of Interest (RoI). The RoI can be defined mathematically. For instance, a rectangular RoI can be specified by two vertical, and two horizontal lines or four points that are the crossing points of those lines. Every line of a rectangular RoI is presented as follows:

(1) $$W_{i}:=\left\{p\in R:p_{y}=m_{i}*p_{x}+b_{i}\right\}$$

where $m_{i}$ is the slope of the $W_{i}$ and $b_{i}$ is a scalar associated with the y-intercept of $W_{i}$. In a rectangular RoI where lines are vertical ($m_{i}=\infty ,x=d_{i}$) and horizontal ($m_{i}=0,y=b_{i}$) the area is defined as $R=\{p\in R:d_{1}<p_{x}<d_{2},\;b_{1}<p_{y}<b_{2}\}$. This paper considers the RoI to be a rectangular area.

### 2.2. Assumptions

We adopt the following commonly used assumptions from the literature [21,22,23] to provide a solid foundation for our study:

- All the sensors in the area are assumed to be mobile sensors and can move freely across the area without any restrictions, i.e., there is no obstacle in the area.
- The sensors are capable of accurately determining their positions within the area and effectively communicating their information to neighbouring sensor nodes.
- The sensors are aware of their environment and can detect when they cross the predefined borders within the designated area.
- In every time step kT, each sensor $s_{i}$ moves toward its proper location. This movement can be shown by Cartesian coordinate, $p_{i}\left(kT\right)$, heading, $\theta_{i}\left(kT\right)$, and the speed, V(kT) for $s_{i}$ at time step kT.
- The values of the aforementioned parameters are defined as ($\theta_{i}\left(0\right)\in \left[0,\pi \right),p_{i}\left(0\right)\in R,V_{i}\left(0\right)=10^{-3}$), during the initial deployment. Within each following time step, every sensor autonomously determines the desired values for its subsequent movement and adjusts its position accordingly.
- The triangular blanket coverage takes place within the network, where sensors exclusively communicate with their neighbouring sensors within their communication range, $r_{c}$. As a result, the movement decisions of each sensor depend on the received location and coordination information from its neighbours at each time step, kT.
- The set of the neighbours for sensor *i* at time step kT, denoted as $N_{i}\left(kT\right)$, comprises those sensors whose distances, $\Delta_{i-j}\left(kT\right)$, are lower than the $r_{c}$, as presented in (2).
  (2) $$\Delta_{i-j}\left(kT\right)=\sqrt{p_{i}{\left(kT\right)}^{2}-p_{j}{\left(kT\right)}^{2}}$$
- The $|N_{i}\left(kT\right)|$ shows the number of neighbours for $s_{i}$ at every time step.
- All sensors possess the capability to detect the boundaries of the Region of Interest and determine the orientations of their tangents. In the case of vertical lines, the assumed orientation is equal to $sin^{-1}\left(m_{i}\right)+\pi /2$.
- The sensing range of every sensor node, $s_{i}$, is limited to $r_{s}$ which is assumed to be lower or equal to $1/\sqrt{3}r_{c}$. Therefore the area every sensor, $s_{i}$, covers, C, is:
  (3) $$C=\{p_{j}\in R:\Delta_{i-j}\left(kT\right)\leq r_{s}\}$$
- In addition, the relation between $V_{max}$ and $r_{c}$ is considered $r_{c}<V_{max}T/\sqrt{2}$ where $V_{max}$ is the maximum velocity of every sensor and T is the period time.
- At each time period T=1, all the mentioned parameters undergo update for every sensor, $s_{i}$.

## 3. Nearest Neighbour Node Deployment Algorithm

The Nearest Neighbour Node Deployment (NNND) algorithm is a distributed algorithm designed for mobile sensor nodes to achieve blanket coverage in an area. Our algorithm incorporates the nearest neighbour rule and control laws proposed in [21,22] to facilitate distributed movements of the sensor nodes.

In the algorithms presented in [21,22], control laws are introduced to achieve triangular blanket coverage in a region defined between two parallel lines, $W_{1}$ and $W_{2}$. By applying the nearest neighbour rule, sensor nodes converge and sequentially move along these lines to cover the Region of Interest. The movement is limited between $W_{1}$ and $W_{2}$ through the use of control laws.

**Figure 1:**
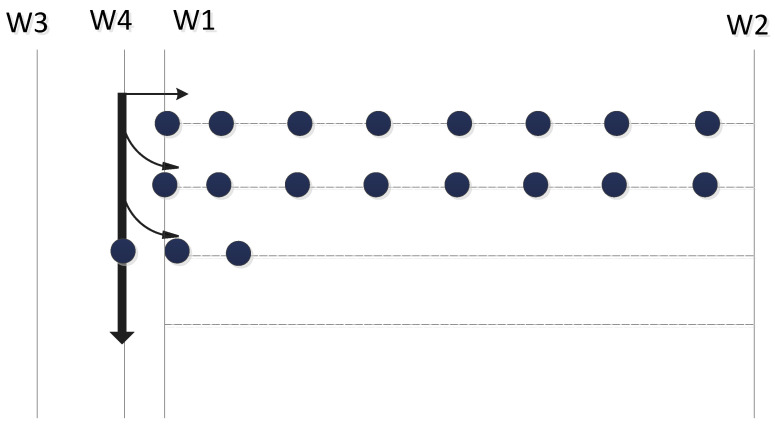
Pattern of sensor movements in NNND algorithm.

To enhance the coverage efficiency, we introduce two additional lines, $W_{3}$ and $W_{4}$, with the same slope as $W_{1}$ and $W_{2}$, to facilitate sensors’ parallel movement. In our NNND algorithm, the sensor nodes begin their movement from line $W_{3}$, where a static leader sensor node is positioned. Each sensor node moves sequentially towards line $W_{4}$ and then transitions towards the unfilled area using newly adopted control laws. This allows the sensor nodes to move in parallel, ensuring full coverage of the area. Figure 1 illustrates a deployment of the NNND algorithm.

The NNND algorithm aims to enable sensors to determine their optimal locations in a distributed manner by exchanging information with their neighbouring sensors, denoted as $N_{i}\left(kt\right)$, at each time step. In the initial deployment phase, the sensors have the initial values of $\theta_{i}\left(0\right),p_{i}\left(0\right)$, and $V_{i}\left(0\right)$ based on the given assumptions. Additionally, the coordination variable $\Theta_{i}\left(kT\right)$, representing the coordination motion of sensor *i*, is initialized at the first step as $\Theta_{i}\left(0\right)=\theta_{i}\left(0\right)$. Furthermore, the sensors can be positioned on different lines at each time step, denoted by γ(i,kT), and can occupy various positions along these lines, represented by $\Gamma (i,\gamma_{i}\left(kT\right))$. At the initial step, for all sensors $s_{i}$, $\Gamma (i,\gamma_{i}\left(0\right))=1$, and γ(i,0)=1. These values are updated at each time step according to the predefined rules to ensure that the sensor nodes are positioned correctly.

Taking into account the initial step and the initial values of the variables, the NNND algorithm utilizes the nearest neighbour rule and control laws to guide the movement of sensors in subsequent time steps. This movement can be projected in two perpendicular directions: (a) the Stabilizer axis and (b) the Thrust-Drag axis. These directions determine the desired trajectory for each sensor. Thus, the new positions of the sensor nodes are updated based on the resultant force acting on each sensor. The resultant force considers factors, such as the influence of neighbouring sensors, environmental conditions, and control laws.

The Stabilizer axis refers to the direction that helps maintain stability and balance in the sensor movement after applying the nearest neighbour rule. It ensures that the sensors stay aligned and coordinated with their neighbouring nodes while navigating through the area. This axis plays a crucial role in achieving a cohesive and synchronized motion among the sensors [14]. On the other hand, the Thrust-Drag axis represents the direction that drives the forward movement of the sensors. The Thrust-Drag axis guides the sensors to move efficiently and effectively towards their target locations within the area.

By considering both the Stabilizer axis and the Thrust-Drag axis, the NNND algorithm enables the sensors to navigate the space while maintaining coordination and making progress towards the goal of achieving full blanket coverage.

### 3.1. Stabilizer Axis

The Stabilizer axis has the same direction $\Theta_{i}\left(kT\right)$ for every sensor $s_{i}$ at time step kT. In a distributed sensor networks with *n* sensors, $S=\{s_{1},s_{2},\cdots ,s_{n}|n\in N\}$, the next direction of sensor $s_{i}$, $\Theta_{i}\left(\left(k+1\right)T\right)$, based on the nearest neighbour rule is equal to $\chi_{i}\left(kT\right)$, Equation (4). The $\chi_{i}\left(kT\right)$ is the average of $s_{i}$ and its neighbouring nodes directions, presented as Equation (5), where $N_{i}\left(kT\right)$ is the set of $s_{i}$ neighbours at the time step kT. Hence, the direction of the Stabilizer axis for each sensor node can vary at every time step kT depending on the directions’ changes.

(4) $$\Theta_{i}\left(\left(k+1\right)T\right)=\chi_{i}\left(kT\right)$$

(5) $$\chi_{i}\left(kT\right)=\frac{1}{|N_{i}\left(kT\right)|+1}\sum_{j\in N_{i}\left(kT\right)+i}\Theta_{j}\left(kT\right)$$

It is an effective and straightforward approach to provide a clear representation of its neighbouring location.

The projection of the neighbouring sensors’ location on the Stabilizer axis is an effective technique for sensor $s_{i}$ to grasp its neighbours’ positions. The $\zeta_{i,j}\left(kT\right)$, represents the projection of the neighbour $s_{j}\in N_{i}\left(kT\right)$ which is calculated in Equation (7). Moreover, ${Ϝ}_{i}\left(kT\right)$ represents the current projection of $s_{i}$ on this axis, Equation (8). The average of these projections is called $\mu_{i}\left(kT\right)$, and is calculated using Equation (9), which represents a proper location for sensor $s_{i}$ based on the nearest neighbour rule at this time step.

(6) $$\zeta_{i,j}\left(kT\right)=\left[cos\left(\Theta_{i}\left(kT\right)\right),sin\left(\Theta_{i}\left(kT\right)\right)\right]*p_{j}{\left(kT\right)}^{T}$$

(7) $${Ϝ}_{i}\left(\left(k+1\right)T\right)=\mu_{i}\left(kT\right)$$

(8) $${Ϝ}_{i}\left(kT\right)=\left[cos\left(\Theta_{i}\left(kT\right)\right),sin\left(\Theta_{i}\left(kT\right)\right)\right]*p_{i}{\left(kT\right)}^{T}$$

(9) $$\mu_{i}\left(kT\right)=\frac{1}{|N_{i}\left(kT\right)|+1}\sum_{j\in N_{i}\left(kT\right)+i}\zeta_{i,j}\left(kT\right)$$

As stated in [22] that all the sensors will be moving in the same direction after a certain number of time steps if the sensor nodes update their locations based on the nearest neighbour rule. However, relying solely on the nearest neighbour rule is only useful when covering a line. Moreover, based on one of the control laws, $\mu_{i}\left(kT\right)$ as a movement point is only valid when sensor nodes are needed in the same line. Therefore, although $\mu_{i}\left(kT\right)$ represents the next point of the movement on the Stabilizer axis, it is not valid when ${Ϝ}_{i}\left(kT\right)>\delta_{i}\left(kT\right)$. The Equation (10) shows the next movement point on the stabilizer axis under this condition where $\delta_{i}\left(kT\right)=\zeta_{i,W_{4}}\left(kT\right)-\left(\frac{\sqrt{3}}{4}s*\left(\gamma_{i}\left(kT\right)-1\right)\right)$.

(10) $${\bar{\mu }}_{i}\left(kT\right)=\left\{\begin{array}{cccc} \; & \zeta_{i,i}\left(kT\right)-\frac{\sqrt{3}}{4} & \; & {Ϝ}_{i}\left(kT\right)>\delta_{i}\left(kT\right)\\ \; & \mu_{i}\left(kT\right) & \; & \;\;otherwise \end{array}\right.$$

The next movement point and the current point present the head and tail of the movement vector. However, the only important value on this axis is the magnitude of this vector at every time step. The velocity value on this axis shows the magnitude of the movement vector and it is calculated as below:

(11) $${\hat{V}}_{i}\left(kT\right)=\frac{{\bar{\mu }}_{i}\left(kT\right)-{Ϝ}_{i}\left(kT\right)}{T}$$

### 3.2. Thrust-Drag Axis

The Thrust-Drag axis constitutes the second component of every sensor, $s_{i}$, movement in the NNND algorithm. The axis name is inspired by the Drag and Thrust forces on the aeroplane that behaves similarly to the final movement vector of $s_{i}$ on this axis.

On this axis, the projections of the neighbouring nodes’ locations, $N_{i}\left(kT\right)$, in the perpendicular direction of the Stabilizer axis is calculated, Equation (12), where $\psi_{i,j}\left(kT\right)$ represents the projection of every $s_{j}\in N_{i}\left(kT\right)$ on the Thrust-Drag axis. Afterwards, the two nearest projected neighbours on this axis to $s_{i}$ which has a greater and lower values than $\psi_{i,i}\left(kT\right)$, the projection of $s_{i}$ on this axis, are considered as $\psi_{i,\beta }\left(kT\right)$ and $\psi_{i,\alpha }\left(kT\right)$, respectively. These two neighbours, alongside the situation of $s_{i}$, determine the next movement point of the $s_{i}$ on this axis. The nearest two neighbours are used to explain the surrounding of the sensor $s_{i}$ and manage its movement across the line.

(12) $$\psi_{i,j}\left(kT\right)=\left[sin\left(\Theta_{i}\left(kT\right)\right),-cos\left(\Theta_{i}\left(kT\right)\right)\right]*p_{j}{\left(kT\right)}^{T}$$

The next movement point for $s_{i}$ on this axis, $\Psi_{i}\left(kT\right)$, is determined based on the following condition:

1. When $s_{i}$ is crossing $W_{1}$ the value of $\Psi_{i}\left(kT\right)$ is calculated as:
  - If only neighbour β exits the value of $\Psi_{i}\left(kT\right)$ is:
    (13) $$\Psi_{i}\left(kT\right)=\frac{\psi_{i,\beta }\left(kT\right)+r_{s}+\psi_{i,i}\left(kT\right)}{2}$$
  - If there is no α and β neighbours and $s_{i}$ is with no neighbour around:
    (14) $$\Psi_{i}\left(kT\right)=\psi_{i,TD.W_{4}}\left(kT\right)$$
    where $TD.W_{4}$ represents an intersection point of W4 and the Thrust-Drag axis at time step kT and the $\psi_{i,TD.W_{4}}\left(kT\right)$ is the projection of that intersection point on the Thrust-Drag axis.
  - And in any other situation:
    (15) $$\Psi_{i}\left(kT\right)=\frac{\psi_{i,\alpha }\left(kT\right)+r_{s}+\psi_{i,i}\left(kT\right)}{2}$$
2. In other situations where $s_{i}$ is not crossing the $W_{1}$:
  - If both neighbour α and neighbour β exist:
    (16) $$\Psi_{i}\left(kT\right)=\frac{\psi_{i,\alpha }\left(kT\right)+\psi_{i,\beta }\left(kT\right)}{2}$$
  - When there is no α and only β neighbour exists for $s_{i}$:
    (17) $$\Psi_{i}\left(kT\right)=\frac{\psi_{i,\beta }\left(kT\right)+\psi_{i,i}\left(kT\right)-r_{s}}{2}$$
  - If there is no neighbour α and $s_{i}$ is the last sensor on the line:
    (18) $$\Psi_{i}\left(kT\right)=\frac{\psi_{i,\beta }\left(kT\right)+\psi_{i,i}\left(kT\right)}{2}$$
  - If there is no α and β neighbours and $s_{i}$ is the last sensor on the line:
    (19) $$\Psi_{i}\left(kT\right)=\psi_{i,i}\left(kT\right)+\frac{r_{s}}{D}$$
    where *D* is a high number relevant to the number of sensors and area to manage the movement of the sensors.
  - And finally when there is no neighbour α and β:
    (20) $$\Psi_{i}\left(kT\right)=\psi_{i,TD.W_{1}}\left(kT\right)$$
  where $TD.W_{1}$ represents an intersection point of W1 and the Thrust-Drag axis at time step kT and the $\psi_{i,TD.W_{1}}\left(kT\right)$ is the projection of that intersection point on the Thrust-Drag axis.
3. There is a specific situation for the second sensor at each line. The specified distance of this sensor from the first sensor at the line makes its $\Psi_{i}\left(kT\right)$ different from other sensors on the line:
  (21) $$\Psi_{i}\left(kT\right)=\frac{\psi_{i,\alpha }\left(kT\right)+\psi_{i,i}\left(kT\right)+\tau }{2}$$
4. Finally, when $s_{i}$ reaches to $W_{2}$:
  (22) $$\Psi_{i}\left(kT\right)=\psi_{i,TD.W_{2}}\left(kT\right)$$
  where $TD.W_{2}$ represents an intersection point of W2 and the Thrust-Drag axis at time step kT and the $\psi_{i,TD.W_{2}}\left(kT\right)$ is the projection of that intersection point on the Thrust-Drag axis.
  The value of $\Psi_{i}\left(kT\right)$ and the current projection of $s_{i}$ on this axis determine the next movement point for the sensor $s_{i}$. The magnitude of this vector is the significance parameter of this vector which will be used in this algorithm. The velocity value, ${\bar{V}}_{i}\left(kT\right)$, the vectors’ magnitude, is calculated as presented below:
  (23) $${\bar{V}}_{i}\left(kT\right)=\left\{\begin{array}{cccc} \; & 0 & \; & i\;on\;the\;line\\ \; & \frac{\Psi_{i}\left(kT\right)-\psi_{i,i}\left(kT\right)}{T} & \; & otherwise \end{array}\right.$$

### 3.3. The Resultant Force

**Figure 2:**
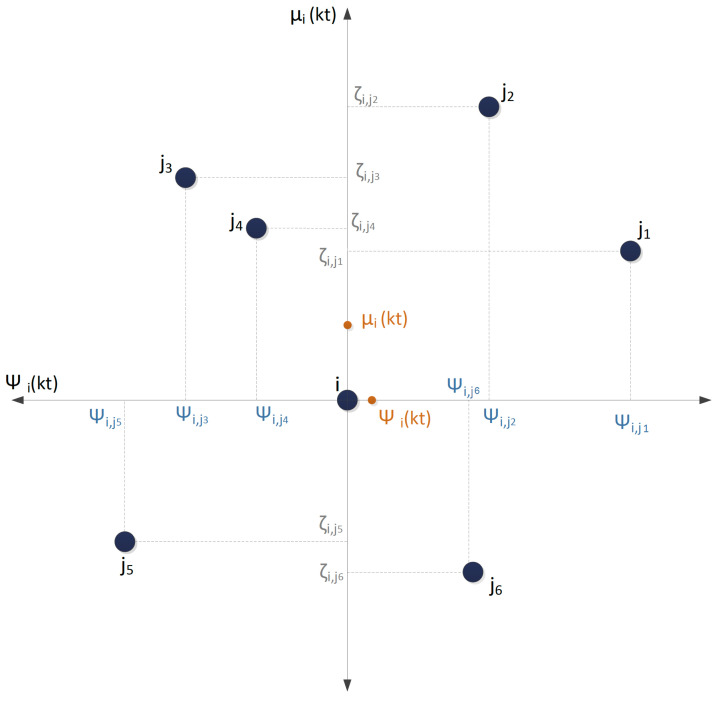
The Thrust-Drag and Stabilizer axis for a sensor $s_{i}$ and its neighbours projections points.

At every time step of the NNND algorithm, every sensor $s_{i}$ in the network moves to a point that is calculated by the resultant force. The resultant force at each step is a vector which consists of both magnitude and direction for every sensor $s_{i}$ to move towards a point. At every time step, kT, for each $s_{i}$ the magnitude of the resultant force corresponds to the final velocity of that sensor. We presented the calculations of the velocity values for both Stabilizer, ${\bar{V}}_{i}\left(kT\right)$, and Thrust-Drag axis, $\bar{V_{i}}\left(kT\right)$, in previous sections and projections of neighbouring sensors are shown in Figure 2. The Equation (24) is utilized to determine the final velocity of every sensor $s_{i}$.

Therefore, the final velocity is calculated as:

(24) $$V_{i}\left(kT\right)=\sqrt{{\hat{V}}_{i}{\left(kT\right)}^{2}+{\bar{V}}_{i}{\left(kT\right)}^{2}}$$

At every time step, sensor $s_{i}$ moves towards a terminal point of a resultant vector with this magnitude.

The direction of the resultant force, $\theta_{i}\left(kT\right)$, for every sensor $s_{i}$ is calculated as below: The Stabilizer axis direction, ϕ(i,kT), is updated based on the situation of the sensors in this time step:

(25) $$\phi \left(i,kT\right)=\left\{\begin{array}{cccc} \; & \Phi & \; & i\;on\;the\;line\\ \; & \Theta_{i}\left(kT\right) & \; & otherwise \end{array}\right.$$

Afterwards, the angle of the resultant force, $\theta_{i}\left(kT\right)$, is adjusted based on the current ϕ(i,kT) as:

(26) $$\theta_{i}\left(kT\right)=\left\{\begin{array}{cccc} \; & \phi_{i}\left(kT\right)+\vartheta_{i}\left(kT\right)-\frac{\pi }{2} & \; & {\bar{V}}_{i}\left(kT\right)\geq 0\\ \; & \phi_{i}\left(kT\right)-\vartheta_{i}\left(kT\right)-\frac{\pi }{2} & \; & otherwise \end{array}\right.$$

where $\vartheta_{i}\left(kT\right)=\cos^{-1}\left(\bar{V_{i}}\left(kT\right)/V_{i}\left(kT\right)\right)$

Finally, the new position of sensor $s_{i}$ at time step kT in the horizontal and vertical axis is calculated based on the angle and the velocity value as:

(27) $$p_{i-x}\left(\left(k+1\right)T\right)=p_{i-x}\left(kT\right)+\cos\left(\theta_{i}\left(kT\right)\right)*V_{i}\left(kT\right)$$

(28) $$p_{i-y}\left(\left(k+1\right)T\right)=p_{i-y}\left(kT\right)+\sin\left(\theta_{i}\left(kT\right)\right)*V_{i}\left(kT\right)$$

In addition to the location of the sensor $s_{i}$ at every time step on different axis, the position of the sensor on each line and the line number that this sensor is located on is important and should be updated for the next time step (k+1)T. If the sensor $s_{i}$ has a neighbour β, the position of the sensor on its line $\gamma_{i}\left(kT\right)$, changes using the Equation (29):

(29) $$\Gamma \left(i,\gamma_{i}\left(kT\right)\right)=\left\{\begin{array}{cccc} \; & \Gamma (\beta ,\gamma_{i}\left(kT\right))+1 & \; & \;only\;\beta \\ \; & \Gamma (i,\gamma_{i}\left(kT\right)) & \; & otherwise \end{array}\right.$$

Also, if the sensor’s position on the line is higher than the maximum expected number of sensors on a line, $\Lambda =\lceil \frac{W_{2-x}-W_{1-x}}{s}\rceil$, and it has crossed the $W_{3}$, the sensor should move to the next line using Equation (30) and the sensor’s position on the new line is set to one as expressed in Equation (31). Obviously, Equation (30) becomes ineffective when movement extends beyond the boundaries of the area. Consequently, the generation of a new line is circumvented.

As mentioned previously, in NNND each stream has a leader recognised as the first sensor node on the line. Utilising multiple leaders in the area; one for each stream; enhances the fault tolerance of the algorithm in comparison to the original algorithm where only one leader; the first sensor node that reaches the first line; is chosen. Losing the first sensor node of the line affects the communication between sensor nodes. However, in NNND failure of one sensor node does not compromise the coverage of the whole area. Moreover, control laws are adjusted to prevent out-of-boundary movements, resulting in a decrease in the total travelled distance by sensor nodes and lower energy consumption in the NNND algorithm. In Table 1, used notations in the NNND algorithm are presented.

(30) $$\gamma \left(i,\left(k+1\right)T\right)=\left\{\begin{array}{cccc} \; & \gamma (i,kT)+1 & \; & i\;on\;the\;line\\ \; & \gamma (i,kT) & \; & otherwise \end{array}\right.$$

(31) $$\Gamma \left(i,\gamma_{i}\left(kT\right)+1\right)=\left\{\begin{array}{cccc} \; & 1 & \; & \Gamma (i,\gamma_{i}\left(kT\right))>\Lambda \\ \; & \Gamma (i,\gamma_{i}\left(kT\right)) & \; & otherwise \end{array}\right.$$

## 4. Performance Study

In this section, we focus on the evaluation of our proposed algorithm, NNND. The NNND is an algorithm under the nearest neighbour category of node deployment algorithms for MSNs. To our best knowledge, the Cheng algorithm stands as the sole counterpart within this category. Thus, we proceed to gauge the performance of NNND by comparing it against the Cheng algorithm.

We begin by outlining the simulation setup, followed by the presentation of the results obtained from the simulations.

### 4.1. Simulation Setup

In this section, the NNND and Cheng algorithms are examined under various configurations. The analysis of these algorithms includes scenarios with different number of sensors and different area shapes, studying the total movement of sensor nodes, runtime, sensing potential and k-coverage. It is important to highlight that within Mobile Sensor Networks, the costs associated with sensing, computing, and communication are relatively insignificant in comparison to the substantial expenses incurred due to mobility. In this context, the energy expended by sensor nodes is predominantly determined by the cumulative distance travelled by all nodes.

The considered formats for the area are: Wide (4 × 1), Square (2 × 2), and Deep (1 × 4). These area formats are chosen to further study the impact of the width and length of the area on sensor movements, sensors’ location and the area coverage. Moreover, for each scenario where area is considered constant we consider a range for the number of sensor nodes in the area to cover the minimum of the minimum and the maximum of the minimum number of sensor nodes for that area. In the following section, the results of our studies for all scenarios are discussed with cR=1 and $sR=\frac{1}{\sqrt{3}}$ using the Matlab R2018b.

### 4.2. Total Movement of Sensor Nodes

**Figure 3:**
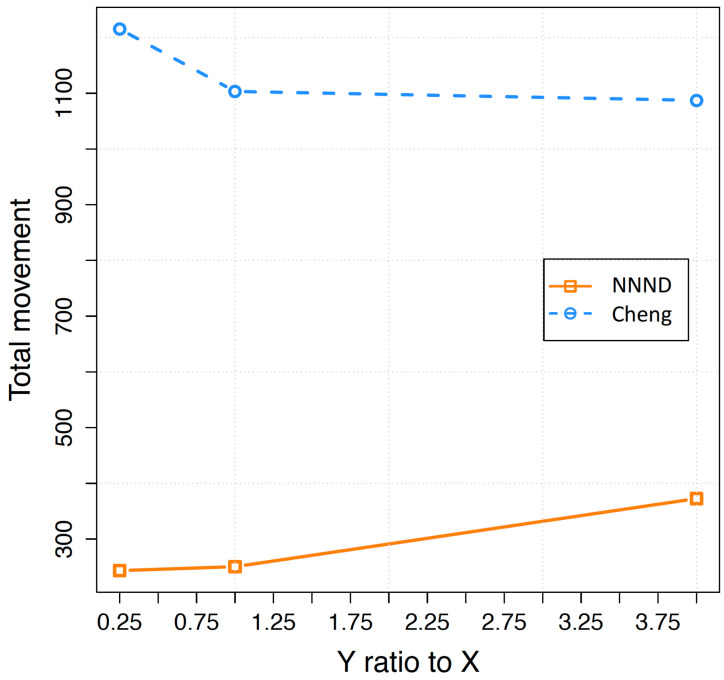
Total movement of 68 sensors based on length to wide ratio of the area.

Figure 3 illustrates the total movement of 68 sensor nodes, which is the maximum of the minimum number of required sensor nodes with the mentioned characteristic for this area, across different area formats for both algorithms. Overall, the NNND algorithm demonstrates significantly reduced travel distance compared to that of Cheng algorithm in all area formats. However, the behaviour of total travelled distance by sensor nodes in different Y to X ratios (different area formats) varies for these algorithms. In the NNND algorithm, the total travel distance by sensors increases as the Y to X ratio increases, while in the Cheng algorithm, this value decreases. To understand the reasons behind these behaviours, we need to consider two aspects. First, we explore why NNND shows a lower travel distance compared to that of Cheng algorithm. Second, we examine why NNND exhibits incremental behaviour while Cheng algorithm demonstrates decremental behaviour as the depth of area increases.

The different behaviours of the NNND and Cheng algorithm in regard to the depth of the area are related to their different principals towards sensor movement at each time step. In an area with *X* width and *Y* length, with *n* number of sensors and sensing range $r_{s}$, the total movement of sensors in Cheng’s algorithm is calculated as:

(32) $$\sum_{j=1}^{\alpha }\sum_{i=0}^{\beta -1}\left(jX-ir_{s}\right)+\left(j-1\right)*y$$

where $\beta =\lceil \frac{X}{r_{s}}\rceil$, $\alpha =\lceil \frac{n}{\beta }\rceil$, and $y=\sqrt{3}/4r_{s}$.

While the total movement of sensors in NNND algorithm is calculated as:

(33) $$\sum_{j=1}^{\alpha }\sum_{i=1}^{\beta -1}(X-(i-1)d)+(j-1)y+\frac{\alpha (\alpha +1)}{2}*y$$

where $\alpha =\lceil \frac{Y}{\sqrt{3}/4}\rceil$$\beta =\lceil \frac{n}{\alpha }\rceil$, $d=\lceil \frac{X}{\beta }\rceil$ and $y=\sqrt{3}/4s$.

To address the first question, an analysis of Equations (32) and (33) can provide insights. In the Cheng algorithm, the total travelled distance is notably higher. This is primarily due to the multiplication of the *X* value by a considerably larger number, which amplifies the total travelled distance. However, in the case of NNND, the total travelled distance is influenced by both *X* and *Y* values. Nevertheless, it is the *Y* values that hold a stronger influence over the final outcome. The described equations verify the behaviour of these algorithms in different areas. It demonstrates that although NNND algorithm has a significantly less total movement of sensor nodes in different shapes of an area, its behaviour is different from the Cheng algorithm as they use different mechanisms for employing sensor nodes.

**Figure 4:**
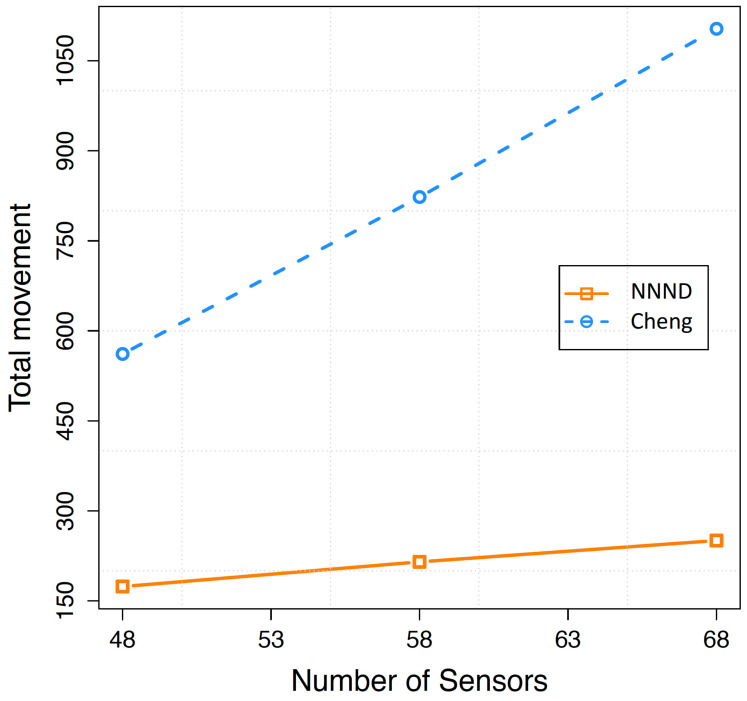
Total movement of different number of sensors in a 2×2 Square area.

In Figure 4, the total movement of 48, 58 and 68 sensor nodes (appropriate range of sensor nodes in this area) for a 2×2 square area is presented. This figure displays the relationship between the total distance travelled by sensor nodes in the area and the varying numbers of sensor nodes. In comparison to the NNND algorithm, the Cheng algorithm demonstrates an overall total movement of sensor nodes that is more than three times higher. As expected, increasing the number of sensor nodes leads to an increased total distance travelled by the sensor nodes in both algorithms. However, the main behaviour difference in this simulation’s results lies in the steeper slope of the Cheng algorithm, indicating a higher growth rate in total movement and wasted energy in comparison to the NNND algorithm when there is a higher number of sensors present in the area.

Finally, the last component of the sensor movement in our simulation study is the wasted walk. As described in the assumptions section of this paper, sensor nodes are able to move freely across the area. Every sensor node in the area can move in four directions. They can move up, down, right and left or a combination of these directions. However, providing blanket coverage requires only specific movements for each sensor node with designated directions from the initial location of the sensor node towards its proper destination. For instance, if a sensor node’s initial location is on the top-left side of the Region of Interest, any movement to the left and up is considered wasteful since it is initially outside the required RoI and does not lead towards the proper location.

**Figure 5:**
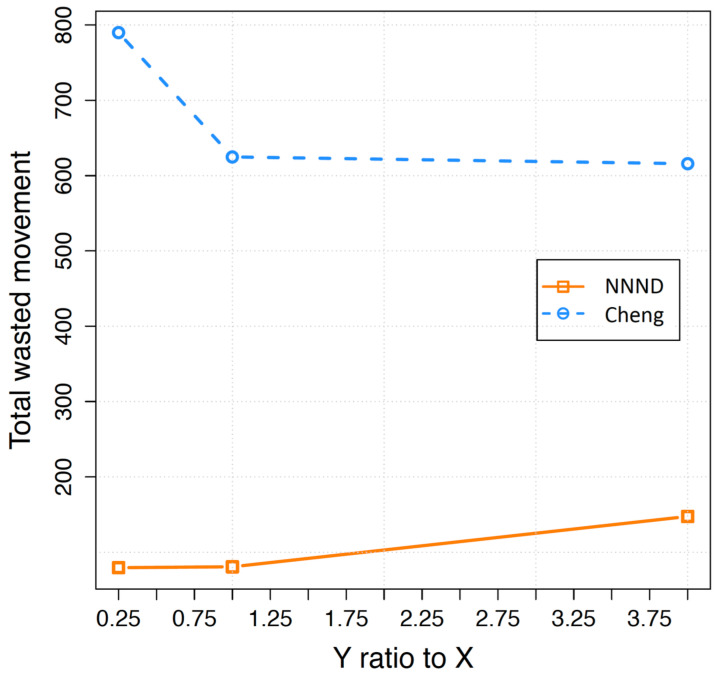
Total wasted movement of 68 sensors based on the length to wide ratio of the area.

While achieving blanket coverage is possible even when not all directions align perfectly, it is crucial to closely examine sensor movements for algorithm efficiency and energy usage optimization. The inefficient movement, which is usually inevitable due to various reasons, is referred to as the wasted walk. Analysing the wasted walk provides a better understanding of the proposed algorithm’s efficiency. In Figure 5, the total wasted walk of 68 sensors in different area shapes is presented. The total wasted walk follows the same pattern as the total movement of sensors shown in Figure 3. Consequently, the total wasted walk increases in the NNND algorithm and decreases in the Cheng algorithm by increasing the Y to X ratio. However, the decremental movement in the Cheng algorithm exhibits a steeper slope than its total travelled distance, while the incremental movement in the NNND algorithm has a lower slope compared to its total movement.

### 4.3. Duration of the Algorithm

**Figure 6:**
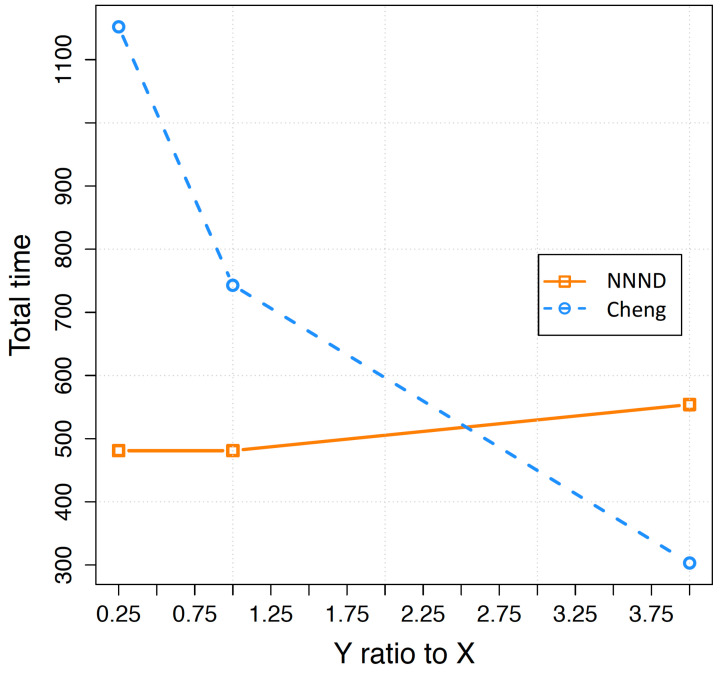
Total consumed time for 48 sensors to be placed in different length to wide ratios of the area.

The total time consumed by an algorithm or the duration of its deployment to locate sensor nodes in their proper locations within the designated area is another parameter that can affect algorithm efficiency. In Figure 6, the total deployment time of the Cheng and NNND algorithms are represented for 48 sensors in different area formats. It is evident from Figure 6, that the consumed time for the NNND algorithm rises as the Y to X ratio increases. This is because a deeper rectangle area requires a higher number of lines to be covered compared to a wider rectangle. In the NNND algorithm, a greater number of sensors have to wait to move to their appropriate lines, resulting in a longer deployment time compared to a wide area.

The higher deployment time for the deeper areas is not a concern in the Cheng algorithm, as sensor nodes move sequentially and do not wait for their turn to be allocated to their lines. However, as shown in the figure, in most scenarios (wider and square shape area) Cheng algorithm requires more time to deploy sensor nodes in the area. The superiority of the Cheng algorithm dominates the parallel movement of sensors in NNND after some point that the Y to X ratio is higher than the 2.5. However, altering the initial location of sensor nodes and rotating the area can easily address the deficiency of the NNND algorithm and transform a deep area into a wide area.

### 4.4. Sensing Potential

**Figure 7:**
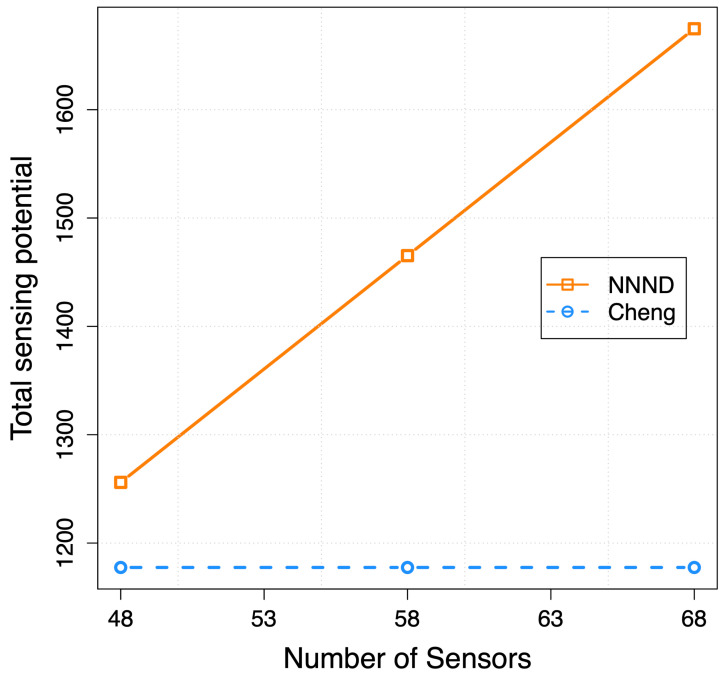
Total potential sensing coverage in the area per number of sensors.

The sensing potential is defined as the total sensing area by existing sensor nodes in the designated area. Generally, the total sensing potential expands by increasing the number of sensor nodes in the area. However, in deployment algorithms such as Cheng and NNND, the total sensing potential depends on the algorithm layout for sensor nodes’ placement. In Figure 7, the relationship between the number of sensors and the total sensing potential is presented.

In both Cheng and NNND algorithms, sensor nodes move in lines with the same static distance between each line. Therefore, they follow the same layout for sensor nodes’ placement. However, the number of sensor nodes on each line is adjustable and varies for different configurations in the NNND algorithm, while it is considered a fixed number in the Cheng algorithm. Based on the existing number of sensor nodes and the length of the area in the NNND algorithm, the number of sensor nodes on each line is calculated as $\frac{N}{Lenght/s*\sqrt{3}/4}$. Therefore, the increase in the number of sensor nodes in the same area results in more sensor nodes on each line and consequently increases the total sensing potential. However, in the Cheng algorithm, any extra sensor node moves forward to the next line and covers the following lines even when the lines are beyond the designated area. Therefore, the potential sensing coverage is always the same for the different number of sensors when full coverage is achieved for the designated area.

### 4.5. K-Coverage

In the Cheng and NNND algorithms, the coverage percentage is not a valid parameter to evaluate the algorithms, as the final coverage of the requested area is always 100% assured. Accordingly, the 1-coverage in deterministic algorithms like Cheng and NNND with enough number of sensors is guaranteed. Therefore, to evaluate the performance of these algorithms exploring the minimum number of sensors that have covered every point of the area, *k* in the k-coverage, is a legitimate interest. The k-coverage demonstrates that every point within the specified area is covered by a minimum of *k* different sensor nodes.

In order to explore the k-coverage of these algorithms, the thermographic snapshots are considered after the deployment of each algorithm is finished. The thermographic snapshot of the final coverage for 64 sensor nodes in the different shapes of RoI with the same area, 4, is used in the following figures to present the coverage for every point in these RoI and understand the layout of sensor nodes in each algorithm. The thermographic snapshot presents 2-coverage points with dark blue and the maximum 18-coverage point by dark red in the RoI. Also, the assigned colour to other the k-coverage is specified as a range in every figure. In the following three thermographic figures for three different forms of area, (wide, square, and deep), are demonstrated.

**Figure 8:**
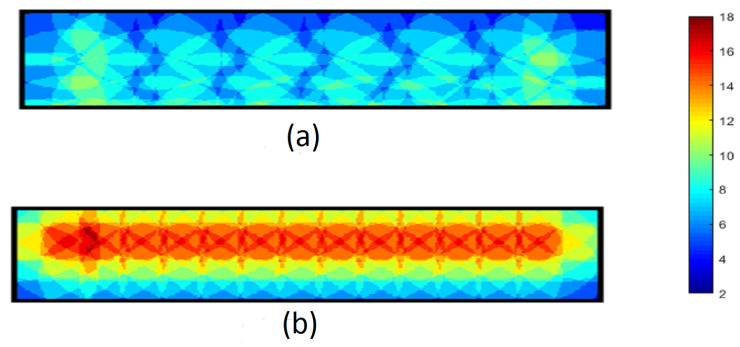
The thermography snapshot of final coverage in a wide area for 64 sensors (**a**) Cheng algorithm (**b**) NNND algorithm.

In Figure 8, the final coverage for 68 sensor nodes in a wide-shaped area is shown. The overall higher k-coverage in NNND Figure 8a is presented by warmer colours in comparison to Cheng’s colder coloured Figure 8b. The reason behind the overall higher k-coverage of the NNND is explained in previous paragraphs. The higher coverage in the left corner of Figure 8a is caused by the close placement of the first and second sensor nodes on the line based on the algorithm requirements for higher reliability. This placement causes a non-symmetric shape in the thermography figure of the NNND algorithm. Although this sensor nodes’ placement’s requirement is the same in both algorithms the different placement of sensor nodes on odd and even lines in Cheng has resulted in a more symmetric k-coverage format in the corners in the area as shown in Figure 8b, in comparison to NNND algorithm which has similar sensor placement for each line.

**Figure 9:**
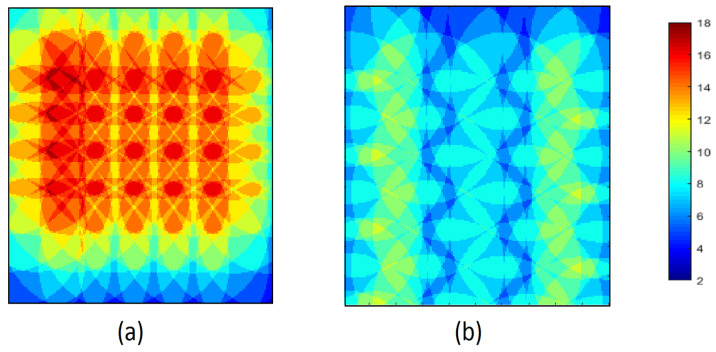
The thermography snapshot of final coverage in a square area for 64 sensors (**a**) Cheng algorithm (**b**) NNND algorithm.

The final deployment of 64 sensor nodes in a square-shaped area is shown in Figure 9. The asymmetric k-coverage thermography of NNND is presented in Figure 9a while the symmetric k-coverage thermography of the Cheng is drawn in Figure 9b. The same pattern as the previous area is shown in this figure for the sensor nodes’ placement. However, the higher number of lines to accommodate the sensor nodes in the requested area depicts the layout differences between these two algorithms more prominently.

**Figure 10:**
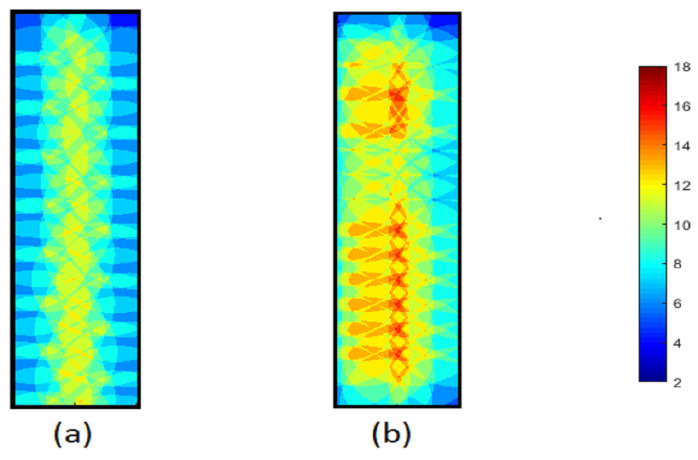
The thermography snapshot of final coverage in a depth area for 68 sensors (**a**) Cheng algorithm (**b**) NNND algorithm.

Although accommodating the higher number of sensor nodes in a requested area is one of the advantages of NNND, this superiority diminishes as the Y to X ratio of an area increases. The increase in depth of the area decreases the number of sensor nodes on every line. The fewer required sensor node on the line decreases the number of additional sensor nodes on each line. Therefore, the average k-coverage in the deeper area is less than in the wide-shaped and square-shaped areas. In Figure 10 the thermographic figures of final coverage for 64 sensors in a deep shape area are shown for (a) Cheng and (b) NNND algorithms. Analysing the k-coverage of Cheng and NNND algorithms in different area shapes while considering the different layouts of sensor nodes in each of those algorithms based on their requirements and strategies confirms the higher overall k-coverage of the NNND algorithm in different scenarios. The higher k-coverage with the same number of sensor nodes depicts the higher utilisation of the existing sensor nodes in the area and reduces the chance of failure.

## 5. Conclusions

Blanket coverage plays a pivotal role in numerous applications within Wireless Sensor Networks (WSNs). It serves as a fundamental service that is essential for the successful operation of various WSN applications. Distributed blanket coverage algorithms, particularly those based on the Nearest Neighbour rule, have emerged as pioneering solutions in Mobile Sensor Networks (MSNs) taking inspiration from the collective movement observed in animal aggregations. However, the proposed algorithm in the literature often suffers from drawbacks. Firstly, sensors tend to move sequentially and at a slow pace, which leads to unnecessary energy consumption due to excessive movements. This inefficiency arises from the lack of coordination and parallelization in their motion. Furthermore, these algorithms often overlook the importance of considering environmental conditions, resulting in sensor movements that extend beyond the intended area. This lack of environmental awareness can compromise the accuracy and effectiveness of blanket coverage in MSNs.

To address these challenges, we have proposed a distributed node deployment algorithm known as Nearest Neighbour Node Deployment (NNND) to provide blanket coverage across the designated area while minimising energy consumption and enhancing fault tolerance. Unlike other algorithms that rely on sequential sensor movements, NNND takes advantage of parallelism by introducing multiple streams of sensor motions, each targeting a specific section of the area. The presence of multiple streams ensures efficient coverage while reducing energy consumption. By adaptively selecting a leader for each stream, the algorithm maintains cohesion and eliminates single points of failure. This approach significantly enhances fault tolerance and incorporates collision avoidance mechanisms, resulting in a network that is more robust and reliable.

Through an extensive performance study, we have compared the final k-coverage, total movement, and time consumed by the NNND against those of the Cheng algorithm. The simulation results confirm the advantages of the NNND, as it achieves enhanced fault tolerance while minimizing energy consumption.

## Figures and Tables

**Table 1:** Notation used in the NNND algorithm.

Notation	Description
$p_{i}\left(kT\right)$	Location of $s_{i}$ at time step kT
$\Delta_{i-j}\left(kT\right)$	Distance between two sensor nodes $s_{i}$ and $s_{j}$
$\theta_{i}\left(kT\right)$	The movement angle of $s_{i}$ at time step kT
$N_{i}\left(kt\right)$	Set of the neighbours for $s_{i}$ at time step kT
$|N_{i}\left(kT\right)|$	Number of neighbours for $s_{i}$ at every time step
γ(i,kT)	Number of the row $s_{i}$ is located on
$\Gamma (i,\gamma_{i}\left(kT\right))$	Position of the $s_{i}$ on the γ(I,kT) row
$\Theta_{i}\left(kT\right)$	Angle between $s_{i}$ coordinate system and Global Cartesian coordinate system
$\zeta_{i,j}\left(kT\right)$	Projection of the location of $s_{j}$ on the S-axis
${Ϝ}_{i}\left(kT\right)$	Current projection of $s_{i}$ on its S-axis
$\chi_{i}\left(kT\right)$	Average of the neighbours’ projections for $s_{i}$ on the S-axis
${\bar{\mu }}_{i}\left(kT\right)$	Ending point of the movement vector for $s_{i}$ on S-axis
$\delta_{i}\left(kT\right)$	The calculated remained distance for $s_{i}$ to take on S-axis
$\psi_{i,i}\left(kT\right)$	Projection of the $s_{i}$ on the TD-axis
$\Psi_{i}\left(kT\right)$	Selected point on TD-axis from the projected points
$\psi_{i,TD.W_{2}}\left(kT\right)$	Projection of the intersection point of the line W2 and TD-axis on the TD-axis
$\psi_{i,TD.W_{1}}\left(kT\right)$	Projection of the intersection point of the line W1 and A-axis on the TD-axis
${\bar{V}}_{i}\left(kT\right)$	Velocity magnitude of $s_{i}$ on TD-axis
$V_{i}\left(0\right)$	Initial velocity value of $s_{i}$
${\hat{V}}_{i}\left(kT\right)$	Velocity magnitude of $s_{i}$ on S-axis
$\theta_{i}\left(kT\right)$	Assigned angle between S-axis and Global Cartesian coordinate

## Data Availability

Not applicable.

## Abbreviations

The following abbreviations are used in this manuscript:

MSN	Mobile Sensor Network
NNND	Nearest Neighbour Node Deployment
WSN	Wireless Sensor Network
TD-axis	Thrust-Drag axis
S-axis	Stabilizer axis
RoI	Region of Interest

## References

[1] Allison C., Hughes C. (1991). Bacterial swarming: An example of prokaryotic differentiation and multicellular behaviour. Sci. Prog. (1933-).

[2] Matsushita M., Fujikawa H. (1990). Diffusion-limited growth in bacterial colony formation. Phys. A Stat. Mech. Its Appl..

[3] Ben-Jacob E., Tenenbaum A., Shochet O., Avidan O. (1994). Holotransformations of bacterial colonies and genome cybernetics. Phys. A Stat. Mech. Its Appl..

[4] Deneubourg J.L., Goss S. (1989). Collective patterns and decision-making. Ethol. Ecol. Evol..

[5] Alt W., Hoffmann G. (2013). Biological Motion: Proceedings of a Workshop Held in Königswinter, Germany, March 16–19, 1989.

[6] Reynolds C.W. (1987). Flocks, Herds and Schools: A Distributed Behavioral Model.

[7] Lei L., Escobedo R., Sire C., Theraulaz G. (2020). Computational and robotic modeling reveal parsimonious combinations of interactions between individuals in schooling fish. PLoS Comput. Biol..

[8] Farine D.R. (2022). Collective action in birds. Curr. Biol..

[9] Herbert-Read J.E. (2016). Understanding how animal groups achieve coordinated movement. J. Exp. Biol..

[10] Rauch E.M., Millonas M.M., Chialvo D.R. (1995). Pattern formation and functionality in swarm models. Phys. Lett. A.

[11] Feinerman O., Pinkoviezky I., Gelblum A., Fonio E., Gov N.S. (2018). The physics of cooperative transport in groups of ants. Nat. Phys..

[12] Balázs B., Vásárhelyi G. Coordinated dense aerial traffic with self-driving drones. Proceedings of the 2018 IEEE International Conference on Robotics and Automation (ICRA).

[13] Vásárhelyi G., Virágh C., Somorjai G., Nepusz T., Eiben A.E., Vicsek T. (2018). Optimized flocking of autonomous drones in confined environments. Sci. Robot..

[14] Vicsek T., Czirók A., Ben-Jacob E., Cohen I., Shochet O. (1995). Novel type of phase transition in a system of self-driven particles. Phys. Rev. Lett..

[15] Savkin A.V. (2004). Coordinated collective motion of groups of autonomous mobile robots: Analysis of Vicsek’s model. IEEE Trans. Autom. Control.

[16] Gao G., Mei Y., Jia Y.H., Browne W.N., Xin B. (2022). Adaptive Coordination Ant Colony Optimization for Multipoint Dynamic Aggregation. IEEE Trans. Cybern..

[17] Jadbabaie A., Lin J., Morse A.S. (2003). Coordination of Groups of Mobile Autonomous Agents Using Nearest Neighbor Rules. IEEE Trans. Autom. Control.

[18] Jiang F., Wang L. (2009). Finite-time information consensus for multi-agent systems with fixed and switching topologies. Phys. D Nonlinear Phenom..

[19] Yu H., Wang Y. (2008). Coordinated collective motion of groups of autonomous mobile robots with directed interconnected topology. J. Intell. Robot. Syst..

[20] Cheng T.M., Savkin A.V. (2011). Decentralized control for mobile robotic sensor network self-deployment: Barrier and sweep coverage problems. Robotica.

[21] Savkin A.V., Javed F., Matveev A.S. (2012). Optimal distributed blanket coverage self-deployment of mobile wireless sensor networks. IEEE Commun. Lett..

[22] Savkin A.V., Cheng T.M., Xi Z., Javed F., Matveev A.S., Nguyen H. (2015). Decentralized Coverage Control Problems for Mobile Robotic Sensor and Actuator Networks.

[23] Savkin A.V., Xi Z., Nguyen H.T. An algorithm of decentralized encircling coverage and termination of a moving deformable region by mobile robotic sensor/actuator networks. Proceedings of the 2013 9th Asian Control Conference (ASCC).

[24] Karimi-Bidhendi S., Guo J., Jafarkhani H. (2022). Energy-Efficient Deployment in Static and Mobile Heterogeneous Multi-Hop Wireless Sensor Networks. IEEE Trans. Wirel. Commun..

[25] Jadbabaie A. (2003). On Distributed Coordination of Mobile Agents with Changing Nearest Neighbors.

[26] Cheng T.M., Savkin A.V. (2009). A distributed self-deployment algorithm for the coverage of mobile wireless sensor networks. IEEE Commun. Lett..

[27] Cheng T.M., Savkin A.V. Decentralized control of a mobile sensor network for deployment in corridor coverage. Proceedings of the 48h IEEE Conference on Decision and Control (CDC) Held Jointly with 2009 28th Chinese Control Conference.

[28] Cheng T.M., Savkin A.V., Javed F. (2011). Decentralized control of a group of mobile robots for deployment in sweep coverage. Robot. Auton. Syst..

[29] Cai X., Wang L., Hui Y., Chen Y., Yue W., Wang H., Zhang Y., Cheng N., Li C. (2023). Coverage Optimization for Directional Sensor Networks: A Novel Sensor Redeployment Scheme. IEEE Internet Things J..

